# Surgical learning curve in reverse shoulder arthroplasty for proximal humerus fractures

**DOI:** 10.1016/j.jseint.2021.07.008

**Published:** 2021-10-09

**Authors:** Leanne S. Blaas, Jian Z. Yuan, Charlotte M. Lameijer, Peter M. van de Ven, Frank W. Bloemers, Robert Jan Derksen

**Affiliations:** a Department of Trauma Surgery, Zaandam Medical Center, Zaandam, the Netherlands; b Amsterdam UMC, location Boelelaan, Department of Trauma Surgery, Research Institute Amsterdam Movement Sciences, Amsterdam, the Netherlands; c Department of Epidemiology and Data Science, Amsterdam UMC, Location Boelelaan, Amsterdam, the Netherlands

**Keywords:** Fracture reverse shoulder arthroplasty, Proximal humerus fractures, Learning curve, CUSUM analysis

## Abstract

**Background:**

Fracture reverse shoulder arthroplasty (fRSA) in geriatric, complex dislocated proximal humerus fractures is becoming the standard treatment next to conservative treatment. fRSA is a multifaceted, reasonably challenging procedure of which functional outcomes and complication rates are likely to depend on the experience of the surgeon. The goal of this study was to determine whether there is a learning curve for fRSA.

**Methods:**

All patients with a dislocated multipart proximal humerus fracture that were treated with an fRSA between 2013 and 2019 in a specialized institution were included. The functional outcomes (Constant Shoulder Score, Oxford Shoulder Score, and range of motion), complications, and operation time of fRSA were assessed with linear regression plots and cumulative summation analysis to establish whether a learning curve was present.

**Results:**

In this cohort study, 50 patients were included. They had a mean age of 77.1 years and were treated with an fRSA by one trauma surgeon. Learning curves were distinguished for functional outcomes, complications, and operation time based on learning targets for daily activity and the mean complications and operation time. Results indicated that an optimal treatment is achieved after performing 20 fRSAs.

**Conclusion:**

The results show that functional outcomes of PHFs treated with an fRSA improve with surgical experience. Also, outcomes are getting less variable after about 20 procedures. Surgeons starting this procedure should be aware of the learning curve and, therefore, should consider guidance from an experienced surgeon to swiftly optimize functional outcomes and prevent unnecessary complications.

The use of the fracture reverse shoulder arthroplasty (fRSA) for proximal humerus fractures (PHFs) has shown reliable and satisfactory results since its introduction.^1^^,^^8^^,^^15^^,^^33^ Several studies have shown better functional outcomes with fRSA than with hemiarthroplasty.^3^^,^^4^^,^^9^^,^^11^^,^^18^^,^^26^^,^^40^ This is largely explained by the medialization of the shoulder’s center of rotation, in which the increased deltoid muscle lever arm compensates for the dysfunctional rotator cuff (Grammonts’ principle).^5^^,^^8^^,^^28^^,^^38^ In addition, the center of rotation is constant which leads to a minimization of shear forces rendering a more stable joint.^38^

Reverse shoulder arthroplasty (RSA) has multiple indications such as osteoarthritis, rotator cuff insufficiencies, fractures, and fracture sequelae. A difference in the treatment of fractures compared to other indications is the need to reattach the tuberosities to the prosthesis. fRSA Is a good option in fracture treatment when preservation of the humeral head is not deemed feasible or has a predicted high chance of avascular necrosis.^1^ The current agreements on indication for fRSA are patients older than 70 years with a dislocated multipart PHF, head-split fractures, and fracture-dislocations.^1^ Osteoporosis, osteoarthritis, and rotator cuff insufficiencies are factors that are considered to attribute to this indication.^1^ Although the fRSA has shown valuable outcomes in PHFs, in literature, it has a high complication rate of 10%-75%.^15^^,^^29^^,^^41^^,^^43^ The most frequent complications are implant instability (1.5%-31%) and reabsorption, nonunion, or malunion of the tuberosities (16%-60%).^6^^,^^14^^,^^22^^,^^24^^,^^25^^,^^35^ In addition, nonunion of the tuberosities can cause subsequent loss of external and internal rotation and subacromial impingement.^7^^,^^25^^,^^36^^,^^39^

Because there are few alternatives when fRSA fails, careful indication and meticulous surgery are paramount.^20^^,^^35^ Potential factors that influence the complication rate are the experience of the surgeon and the proper indication for surgical treatment.^21^^,^^27^^,^^28^^,^^31^^,^^35^^,^^43^ Kempton et al described a decrease in complication rate after performing 40 RSAs.^28^ This learning curve showed an association between diminished complication rate and an increase in the number of surgeries performed. More recently, a steeper learning curve was presented in which the complication rate and operation time diminished after performing only 15 RSAs.^15^^,^^23^ In the latter two studies, none of the patients received an RSA for a PHF. Several other studies mention that the experience of the surgeon potentially influences the functional outcome; however, they did not objectify this with data.^1^^,^^23^^,^^27^

A learning effect consists of two separate modalities: improvement in functional outcomes and decrease of outcome variability. First, there is the learning curve that shows whether there is an improvement in time with regard to (functional) outcomes (eg, improved range of motion [ROM], less complications). The other type of learning curve assesses the decrease in variability of outcome measures and shows whether, after a certain number of procedures, the outcomes contain less outliers (eg, more consistent results). Although learning curves are identified in RSA treatments for various indications such as rotator cuff arthropathy and osteoarthritis, to our knowledge, there is no sufficient scientific evidence to support the existence of a learning curve for PHFs treated with fRSA. As fRSA in many respects is different from “regular” RSA, and considered more difficult than performing an RSA in some respects, the need for a study specifically for fRSA performance was established. Especially for the countries where the treatment of (proximal humerus) fractures is performed by trauma surgeon and orthopedic surgeons. The goal of this study is to assess whether a learning curve can be determined in a cohort of patients treated with an fRSA for PHFs.

We hypothesize that the functional outcomes and complication rate after fRSA will improve with increased experience of the surgeon. In PHFs compared to other indications, the anatomical landmarks are less well-defined. In addition, the management of the fractured tuberosities is often challenging. Therefore, we also hypothesize that the learning curve in fRSA treatments might be less steep, and the resulting learning phase might be longer than that reported in “regular” RSA. The aim of this study was to (1) assess functional outcome and (2) assess complication rate with learning curves of fRSA placement for PHFs.

## Materials and methods

In this cohort study, patients were included with multipart, dislocated PHFs that were treated with an fRSA in Zaandam Medical Center (Zaandam, The Netherlands), a referral center for complex PHFs and their sequelae. Patients were treated between 2013 and 2019. All procedures were performed by a well-rounded trauma surgeon who had no prior experience with RSAs. All enrolled patients were treated with the Affinis Fracture Inverse Shoulder System by Mathys Medical. The fractures were classified according to the Neer classification by one trauma surgeon and the first author. All patients signed informed consent.

Criteria for inclusion were patients treated with an fRSA with at least one-year follow-up, both in acute PHF cases as well as after a failed attempt at conservative treatment. To be included in this study, patients had to have a combination of two of the following criteria for fRSA: aged over 70 years, 3- or 4-part fracture, preexistent osteoporosis, head-split, or fracture-dislocation.^1^ Patients with a language barrier, dementia, or neurological disorders of the upper extremities were excluded. The study protocol was assessed by the regional medical ethics board and was approved.

### Surgical procedure

The Affinis Fracture Inverse (Mathys Ltd., Bettlach, Switzerland) was implanted through a deltopectoral approach, and the patients were in beach-chair position. Surgery was performed under general anesthesia in combination with an interscalene brachial plexus block. During the procedure, a tenotomy of the long head of the biceps was performed. The baseplate was fitted to border the inferior part of the glenoid to minimize scapular notching. No additional tilting was performed. The tuberosities were fixated with sutures, and the supraspinatus tendon and subscapularis tendon, if preserved, were fixated with a MaxBraid (Zimmer Biomet, Warsaw, IN, USA). A few patients were treated with a Supercable (Kinamed), a synthetic cerclage wire with a metal locking mechanism, used for the adherence of the tuberosities. However, some patients experienced discomfort due to the locking mechanism, and therefore, the surgeon switched to the NICELOOP (Wright Medical, Memphis, TN, USA) as a cerclage “wire” through both the anterior and posterior rotator cuff insertions and the designated hole in the neck of the prosthesis.

### Functional outcomes—patient-reported outcome measures and range of motion

To assess the functional outcome, patient-reported outcome measures (PROMs) and ROM were assessed. Two questionnaires were administered with a minimum of one-year follow-up: the Constant Shoulder Score (CSS) and the Oxford Shoulder Score (OSS).^16^^,^^17^ Furthermore, the ROM of the shoulder was measured with a goniometer. To avoid interobserver bias, all outcomes were collected by the same researcher. In addition, patient characteristics were collected from patient files. The results were assessed by the lead author (L.S.B.) with support from a statistics expert (P.M.v.d.V.).

### Complications

For the assessment of the complication rate and the number of revisions, data were retrieved from patient files. Complications were categorized into minor and major complications. Minor complications were classified as complications that do not require considerable revision surgery or longtime medication use. Complications where revision surgery or long-term antibiotic use were necessary and the outcomes of surgery were compromised were classified as major complications.

### Operation time

Finally, operation time was also recorded as an outcome measure. Operation time was defined as the time from incision to closure of the wound in minutes. Detailed operation times were retrieved from the patient files.

### Statistical analysis

Descriptive statistics were used to summarize patient and fracture characteristics. Categorial variables were described by frequency and percentage, and continuous and ordinal variables by their median and interquartile range (IQR). Results were visualized using scatter plots to assess whether a clear trend in the effect of the cumulative number of surgeries on the outcome measures was present. A regression line was added to the scatter plots for assessment of a linear trend. To determine a learning curve based on these outcomes, an additional learning curve analysis was performed, the cumulative summation (CUSUM) analysis. The linear regression plots and CUSUM are complementary visualization of the outcomes. The linear regression plot renders trends ascertaining improvement of the outcomes, while the CUSUM is an analysis that compares outcomes relatively to each other in order of treatment to objectify whether outcomes become more consistent (closer to a predefined target level).

A CUSUM analysis was performed for functional outcomes (PROMs and ROM), complication rate, and operation time in fRSA treatments. This statistical analysis represents the most appropriate statistical method for evaluating a learning curve of a surgical procedure.^42^^,^^44^ The CUSUM analysis continuously compares performance of an outcome to a predefined target level. The resulting cumulative sum curve graphs from this analysis show on the horizontal axes all consecutive patients in a chronological order. The vertical axes show the cumulative performance on the metric, compared to the target level.^42^^,^^44^ For nonbinary metrics, the CUSUM decreases by the absolute difference between target level and the performance level if the performance level is worse than the target level.^44^ Conversely, the CUSUM increases for nonbinary metrics by the absolute difference between the performance level and the target level if the performance level is better than the target level. This CUSUM chart then allows for a visual analysis of the cumulative performance versus target over the performed treatments.^42^^,^^44^ A learning curve can be determined when the cumulative sum curve indicates a downward trend in the first phase, also called learning phase, while an increasing trend can be determined in a secondary phase, also called consolidation phase. In a final third phase, also called the mastering phase, performance reaches an optimal steady level.^37^

The target levels for the different metrics for the determination of the learning curves for the functional outcomes were defined based on current literature and expert opinion.^44^ The OSS has predefined levels to support the interpretation of the outcomes.^17^ Based on the predefined levels, a target level of 34.5 was chosen. For the CSS, the target was set at 50 based on the studies by Booker et al and Alta et al.^2^^,^^10^ For the ROM, targets were based on the activities of daily living in the elderly population (eg, eating, washing under armpits, combing hair). Therefore, the following targets were set: forward flexion 105°, extension 40°, abduction 90°, and external rotation 15°.^19^^,^^34^ The target level for the learning curve of the complication rate was transitioned to a binary outcome of being either with or without complication. The mean of the operation time was the target level for this outcome measure.^30^ SPSS IBM 24 (IBM, Armonk, NY, USA) was used for the statistical analysis. The CUSUM analysis was performed using R 2020 (The R Foundation for Statistical Computing) (R-script is available on request to the lead author.).

## Results

**Figure 1:**
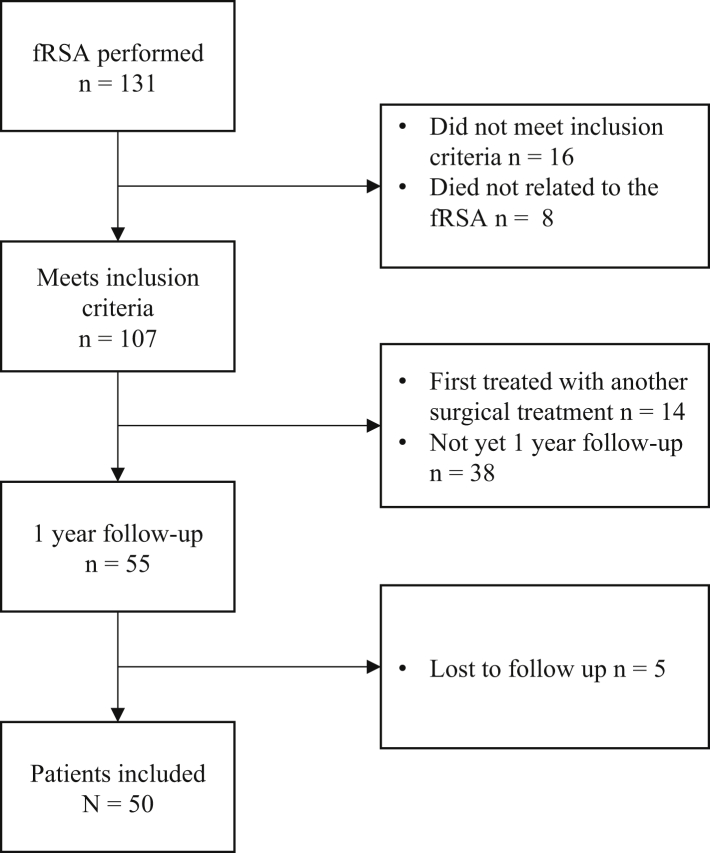
Flow chart of patient inclusion.

A total of 50 patients were included (46 female, 92%) with a mean age of 77.1 years and a minimum follow-up duration of 1 year (median 14.5 months; IQR [13.00, 19.25]). Figure 1 shows the flowchart of the included patients, and the baseline characteristics of the cohort are shown in Table I. Results of the performance assessment in this patient cohort are divided into results for functional outcomes and operation time (Table II).

**Table I:** Baseline characteristics.

Variables∗	Total (N = 50)
Sex, female	46 (92%)
Age, yr	77.1 (9.15)
ASA classification	
1	4 (8%)
2	28 (56%)
3	18 (36%)
Anticoagulation use	20 (40%)
Neer classification	
1 part	1 (2%)
2 part	16 (32%)
3 part	20 (40%)
4 part	13 (23%)
Headsplit	16 (32%)
Fracture-dislocation	8 (16%)
Tuberosity healing, yr	44 (88%)

*SD*, standard deviation; *ASA*, American Society of Anesthesiologists.

∗ Continuous data are presented as mean (SD) and categorical data as number of patients (percentage of group of patients).

**Table II:** Postoperative outcomes and operation time.

Variables	fRSA
PROMs	
OSS	37.0 [30.5, 43.5]
CSS	59.0 [40.0, 70.3]
ROM	
Forward flexion	105.0 [81.8, 132.0]
Extension	45.0 [31.0, 52.0]
Abduction	93.0 [77.3, 111.8]
External rotation	16.0 [4.0, 30.0]
Operation time	125.5 [111.3, 155.3]

*fRSA*, fracture reverse shoulder arthroplasty; *PROM*, patient-reported outcome measures; *OSS*, Oxford Shoulder Score; *CSS*, Constant Shoulder Score; *ROM*, range of motion; *IQR*, interquartile range.

Data are provided as median with [IQR].

### Functional outcomes—PROMs and ROM

**Figure 2:**
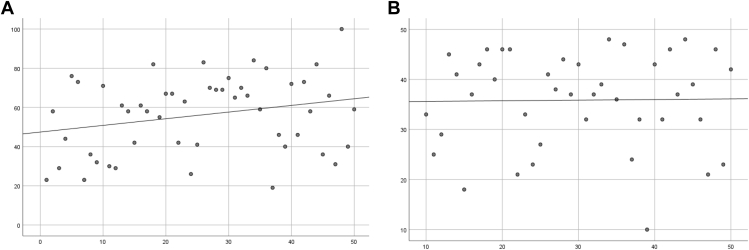
Functional outcomes—*PROMs***(A)** CSS (R^2^ = 0.064) and **(B)** OSS (R^2^ = 0.000). *PROM*s, Patient-Reported Outcome Measures; *CSS*, Constant Shoulder Score; *OSS*, Oxford Shoulder Score.

**Figure 3:**
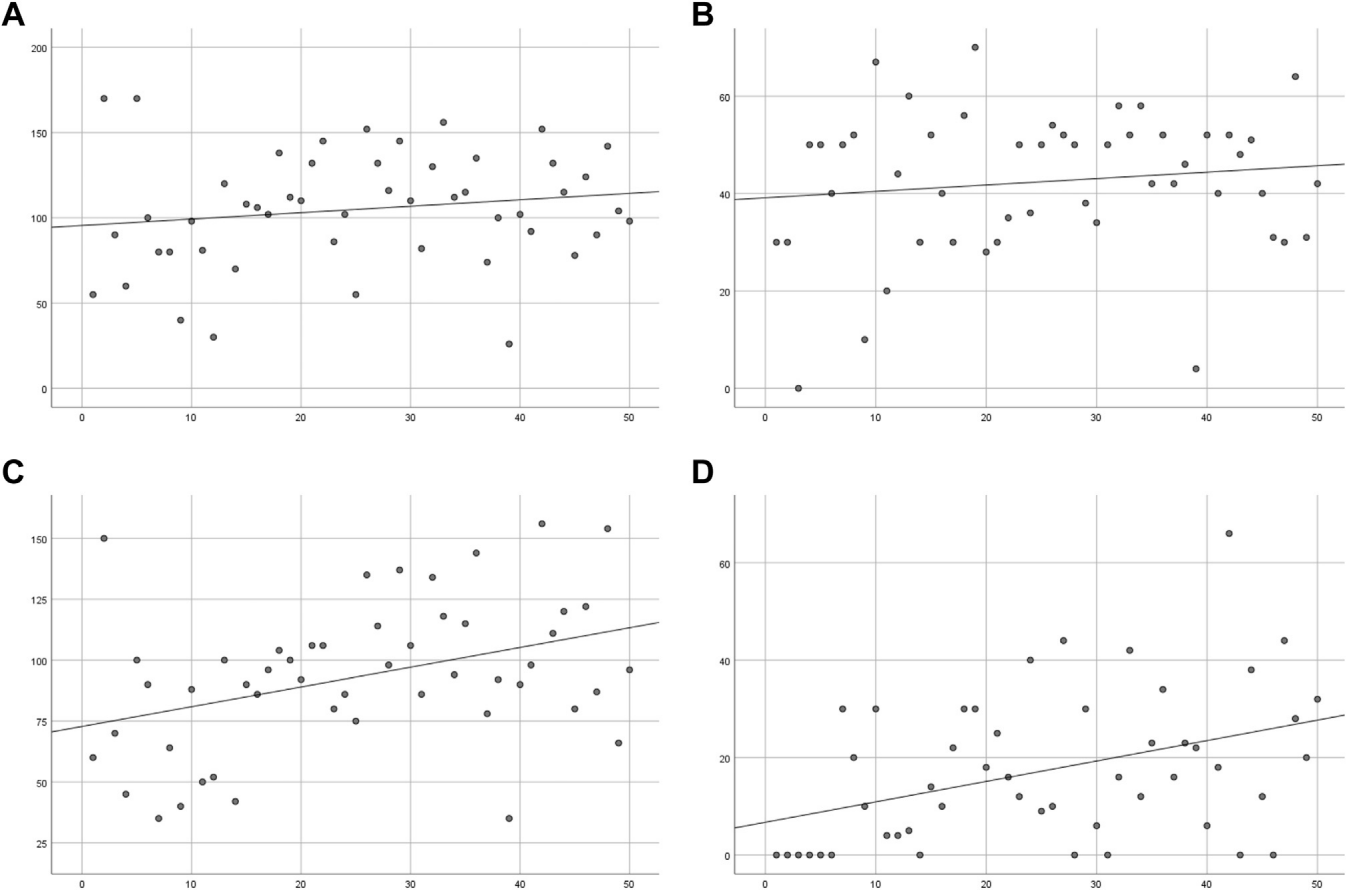
Functional outcomes—*ROM***(A)** forward flexion (R^2^ = 0.027); **(B)** extension (R^2^ = 0.017); **(C)** abduction (R^2^ = 0.150); and **(D)** external rotation (R^2^ = 0.162). *ROM*, range of motion.

Figure 2 shows the linear regression plots of the PROMs, and Figure 3 of the ROM. Within the PROMs, the OSS does not show relevant improvement. However, the CSS does show a clear trend of improving functional outcomes with increasing numbers of patients treated with fRSA. The explained variance is small, which indicates that although there is improvement on average, the outcome measures have a high variability. The abduction and external rotation show a clear trend of improvement over time. The forward flexion and extension also show an improvement, however, with a slightly smaller trend.

**Figure 4:**
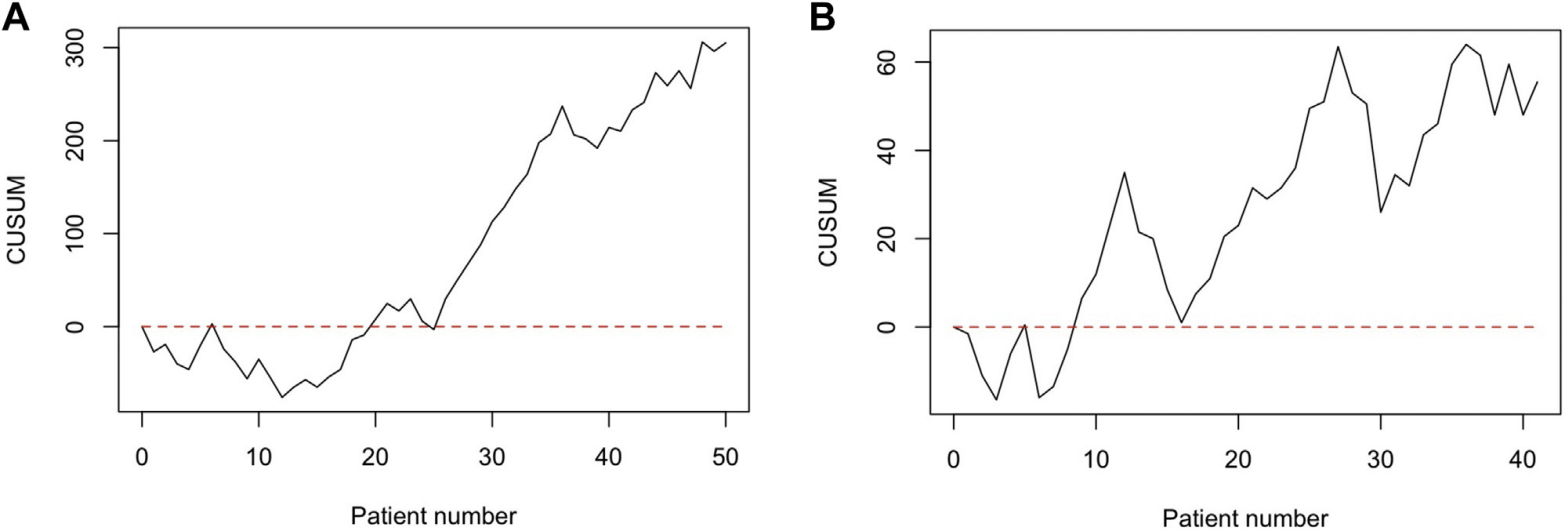
Cumulative sum curve for metrics related to functional outcomes assessed with the questionnaire: *PROMs***(A)** CSS, target 50; **(B)** OSS, target 34.5. *PROMs*, Patient-Reported Outcome Measures; *CSS*, Constant Shoulder Score; *OSS*, Oxford Shoulder Score.

The cumulative sum curves of outcomes related to the PROMs assessed with the questionnaires are shown in Figure 4. The cumulative sum curve for the CSS (Fig. 4, *A*) and OSS (Fig. 4, *B*) indicate the existence of a learning curve. Two phases can be distinguished, a first phase which can be indicated as the learning phase, and a second phase which can be indicated as the consolidation phase. A cutoff point for performance level of CSS was reached after 15-20 treatments, and that of OSS after 20-25 treatments. This indicates that the learning phase takes approximately 20 cases.

**Figure 5:**
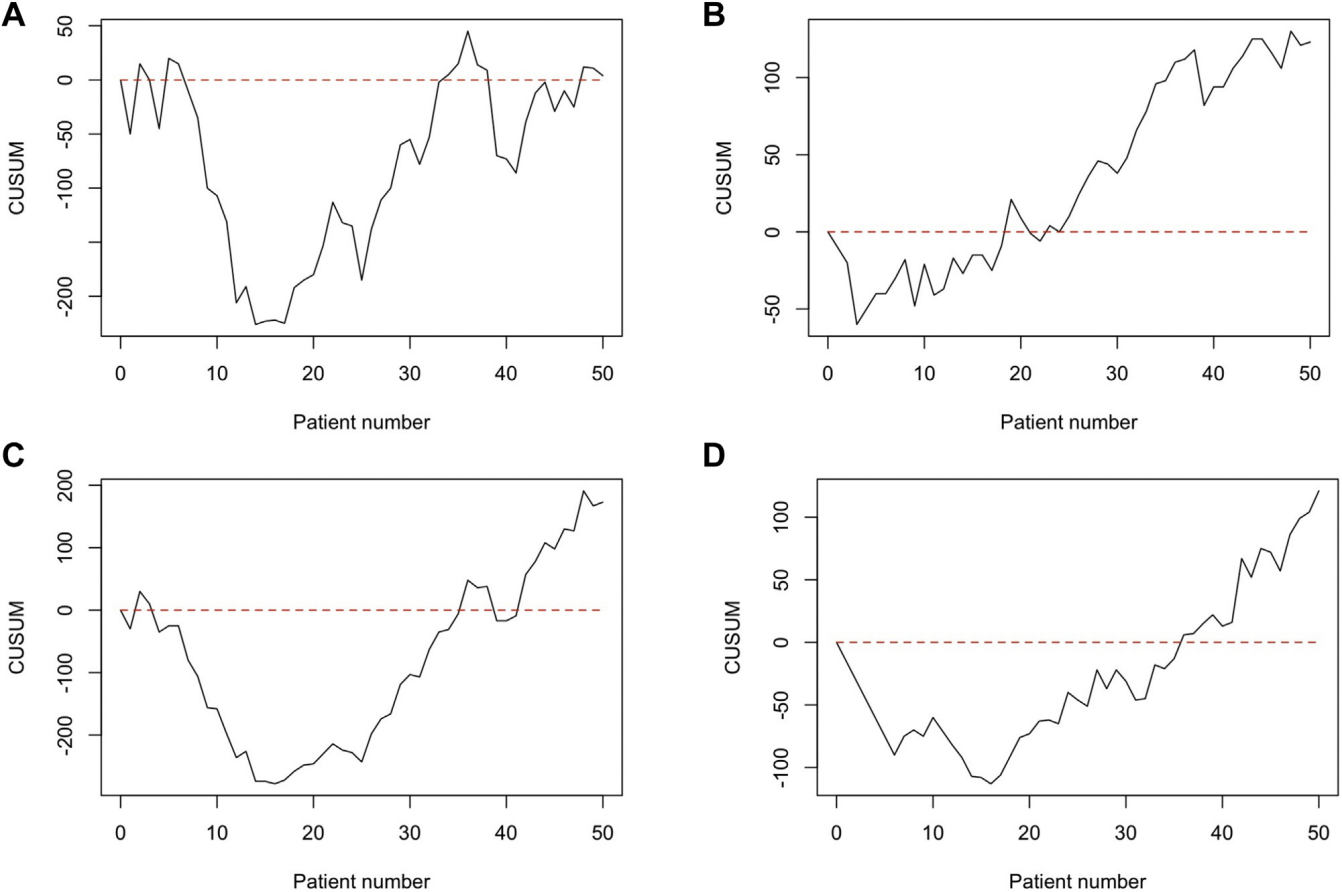
Cumulative sum curve for metrics related to functional outcomes assessed with the goniometer: *ROM***(A)** forward flexion, target 105°; **(B)** extension, target 40°; **(C)** abduction, target 90°; and **(D)** external rotation, target 15°. *ROM*, range of motion.

The CUSUM curves for the ROM after an fRSA at one-year follow-up are shown in Figure 5. The forward flexion (Fig. 5, *A*) and extension (Fig. 5, *B*) show three phases. The first phase which can be indicated as learning phase, a second phase which can indicate a consolidation phase, and a third mastering phase. A clear cutoff point between the learning phase and consolidation phase for forward flexion is difficult to objectify, but it is suggested that this cutoff point is around 15-25. For extension, a cutoff point can be distinguished around 20 treatments. The mastering phase can be distinguished at approximately 40 treatments for the forward flexion as well as for the extension. For the abduction and external rotation, only the learning and consolidation phases can be distinguished. The cutoff points between these phases, when the target performance was reached, can be seen at 25 treatments for the abduction and at 20 treatments for the external rotation.

### Complication rate

The average complication rate of our study cohort was 20% (Table III). Out of the 50 patients, six patients had a minor complication (12%) of which three had a nerve palsy which resolved itself over time. The other three had complaints due to the lock of the Supercable (Kinamed), a synthetic cerclage wire with a metal locking mechanism, used for the adherence of the tuberosities. After consolidation of the tuberosities in these patients, the cable was surgically removed. Three (6%) patients had a major complication (Table III). Of these three, one patient had complaints fitting subacromial impingement, which was due to a high adherence of the greater tubercle to the prosthesis in combination with a Bigliani type 3 acromion. Therefore, a subacromial decompression and acromioplasty was performed. The second patient had a shoulder dislocation postoperatively. The third patient had an aseptic loosening of the humeral stem for which revision ensued. Finally, two patients fell on their shoulder postoperatively. One of those patients had a periprosthetic fracture and needed an open reduction and internal fixation. The other patient had a shoulder dislocation but did not experience any pain and refused further surgery. As the periprosthetic fracture and the shoulder dislocation were unrelated to the initial prosthesis placement, these two were not considered a complication of fRSA placement.

**Table III:** Postoperative complications after fRSA.

Complications	Time from surgery	Treatment and outcomes
Minor		
Radial nerve palsy	Postoperative	Complete recovery after 6 weeks
Axillary nerve palsy	Postoperative	Persistent paresthesia after 12 mo
Radial nerve palsy	Postoperative	Complete recovery after 6 weeks
Pain supercable	7 mo	Removal supercable
Impingement supercable	11 mo	Removal supercable
Pain supercable	8 mo	Removal supercable
Major		
Impingement	3 mo	Subacromial decompression and acromioplasty
Loosening humeral stem, aseptic	7 mo	Revision
Shoulder dislocation	1 mo	Open shoulder reduction

*fRSA*, fracture reverse shoulder arthroplasty.

**Figure 6:**
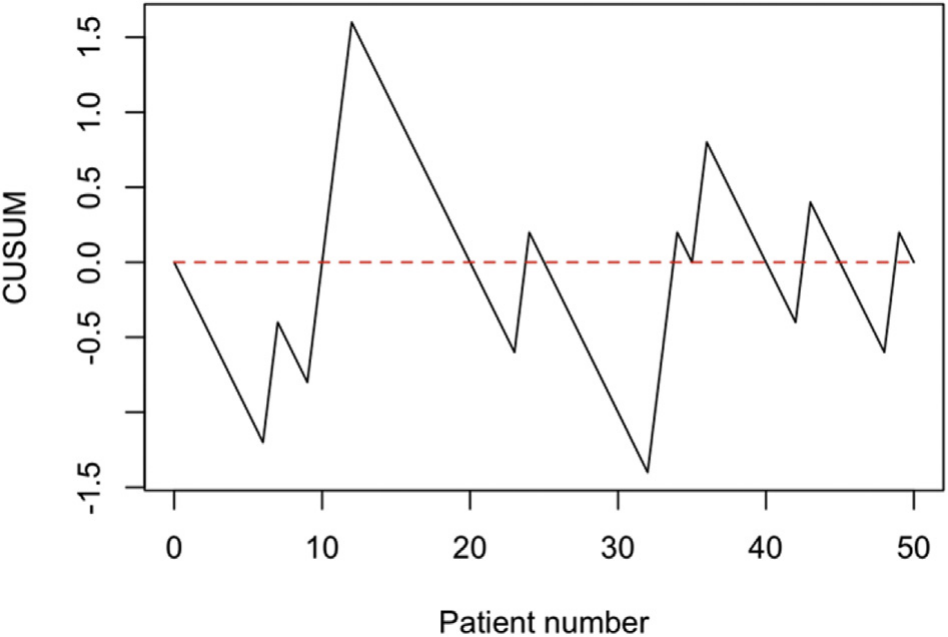
Cumulative sum curve for metrics related to complications: CUSUM based on probability of getting a complication with a probability of 0.20. *CUSUM*, cumulative summation.

The CUSUM analysis of the complication rate shows a learning curve relative to the probability of reaching a target level of the mean complication rate of the cohort (20%) after 12 fRSAs (Fig. 6).

### Operation time

**Figure 7:**
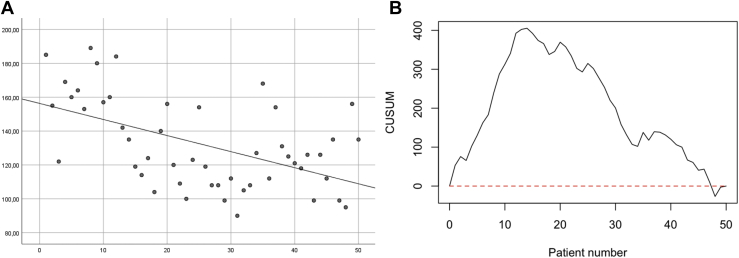
Operation time: **(A)** The scatterplot (R^2^ = 0.275) and **(B)** the CUSUM chart with the mean as target. *CUSUM*, cumulative summation.

The mean operating time of our cohort was 132 minutes (standard deviation 26.39). The CUSUM plot first shows a downward trend where the operation time exceeds the mean. Figure 7 shows the scatterplot and CUSUM of the operation time. Shorter operation times were observed after 10 to 20 patients.

## Discussion

To our knowledge, this study is the first to determine a clear learning curve in the treatment of PHF with an fRSA. Previous studies proving learning curves in orthopedic and trauma surgery presented cutoff points to be reached between 10 and 40 for RSA treatment with a variety of indications.^15^^,^^23^^,^^28^^,^^43^ Our target levels, between 12 and 25 for the various outcome measures, are comparable. More specifically, we found learning cutoff points for functional outcomes between 15 and 25, after 12 fRSAs for complication rates and after 10-15 fRSAs for operation time. In contrast to the literature, we studied the learning effect of fRSAs for PHFs, for which we hypothesized that the learning phase would be longer. However, our results are in accordance with the range of the learning effect as found in the literature.^15^^,^^23^^,^^28^^,^^43^

Comparing our findings to the literature regarding the learning curve of RSAs, our results show similar learning cutoff points.^15^^,^^23^^,^^28^^,^^43^ With regard to the functional outcomes, only Hasan et al described functional outcomes with regard to a learning curve in a group of patients treated for rotator cuff deficiencies, arthritis, or a revision after hemiarthroplasty.^23^ The authors described the simple shoulder test, American Shoulder and Elbow Surgeons score, and ROM.^23^ They found a learning effect at 15 to 20 patients by splitting their cohort into two groups, the first 15 patients as one group, and the remaining 45 patients as a second group.^23^ Our learning cutoff points for functional outcomes are between 15 and 25, which is slightly above their targets. Potentially this could be explained by the required reattachment of the tuberosities in our cohort with only PHFs, as this technique might add to the length of the learning phase.

All literature on learning curves for RSA contains the assessment of a learning curve for the complication rate.^15^^,^^21^^,^^23^^,^^28^^,^^31^^,^^43^ Of these, three studies found a learning effect in 7, 15, and 40 treated patients, respectively.^23^^,^^28^^,^^43^ These results show a large variability regarding a learning cutoff point. In addition, the other 3 studies did not find a learning effect.^15^^,^^21^^,^^31^ This may indicate that the complication rate alone may not be a sufficient outcome measure for assessing learning effect.

A learning curve for the operation time was only established by Choi et al.^15^ The learning effect for operation time was calculated to be at 15 patients, which is similar to our 10 to 15 patients. Shortening the operation time has many benefits such as less blood loss and infection but can also correlate with worse functional outcomes.^13^^,^^30^ Therefore, by studying a learning effect of a surgical treatment, it is beneficial not only to assess operation time but also to consider other outcome measures.

### Strength and weaknesses

In comparison to the aforementioned literature studying learning curves, our methodology contains several improvements. Both with regard to the analyzed outcome measures as well as to the applied statistical method, a more extensive approach was used. In addition to the complication rate and operation time as outcome measures, we included the functional outcomes which are generally considered to be the most important outcome measures for patients. Adding functional outcomes to the operation time and complication rate, therefore, provides a more comprehensive and nuanced view on the actual surgical performance.

Also, the applied statistical method has not been used before for determining the learning curve in fracture treatment. The CUSUM method determines the learning curve based on the respective variability between individual treatments relative to a transparent determined target level. This method yields a more detailed view of the performance of the surgeon compared to a method that assesses the performance of two relatively arbitrary divided groups (as has been used as a method before^21^^,^^28^^,^^43^). The CUSUM method is, therefore, favorable given the ability to visualize specific changes which can be easily quantified.^42^ Recent studies assessing learning curves regarding surgery have been using this method which could indicate that it is becoming the new standard methodology to determine learning curves.^30^^,^^32^

The learning curves on performance improvement (linear regression plots) all show an improvement of the direct measured outcomes except for the OSS. This is explained by the small IQR (30.5-43.5) of the OSS. The reason for this small range can be found in the fact that the OSS is solely based on patient-reported outcomes, whereas the CSS also encompasses the ROM.^16^^,^^17^ As the CSS improves together with the ROM over the number of performed surgeries, one could reason that patient-related outcomes, as they are measured in the OSS, are not influenced by the experience of the surgeon, and patients report a satisfactory joint function with the fRSA.

To determine the learning curves, target levels have to be defined. Instead of using the mean as a target to determine the learning curve,^37^ we used preset target levels from literature when possible. Using the mean of the patient cohort would only indicate relative performance of the surgeon to him or herself instead of relative to a reasonable preset target level. Therefore, using preset target levels gives a better indication of the surgeon’s performance.^12^^,^^42^^,^^44^ Those preset target levels were set to plausible levels based on relevant literature and expert opinion.^2^^,^^10^^,^^17^^,^^19^^,^^30^^,^^34^ Literature on requirements for daily activities has not been used because it studied the ROM based on young, healthy participants and used the maximum needed ROM. The target levels for the ROM in this study were set to reasonable levels that would enable these elderly patients to perform their daily activities.

In our study, we excluded patients with fracture sequelae (patients with malunion or nonunion after another surgical treatment) because literature shows that there is a higher complication rate for treatment with fRSA in this group as well as decreased functional outcomes. Enrolling these patients in the study would potentially “blur” the (statistical) picture and refrain us from determining an accurate learning curve.

### Clinical implications

This study proves the existence of a learning curve for the fRSA with optimal results achieved after 20 procedures. Given this number of procedures required to provide patients with optimal care, it is important for surgeons to have support from skilled colleagues during approximately 20 procedures. Furthermore, fRSA shows overall good clinical results and is, therefore, a valuable option in the treatment of PHFs.

## Conclusion

Surgical treatment of PHFs with fRSAs improves with surgical experience during the learning phase of 20 procedures. This learning effect was seen for all assessed outcome measures. Surgeons starting this procedure should be aware of the learning curve and should consider guidance from a senior surgeon to optimize functional outcomes and prevent unnecessary complications associated with inexperience.

## Disclaimers

*Funding:* This research was performed as a proximal humerus fracture project and as such supported by an unrestricted educational grant by Mathys Medical Ltd.

*Conflicts of interest:* L.S. Blaas has an unrestricted educational grant from Mathys Medical Ltd. The other authors, their immediate families, and any research foundation with which they are affiliated have not received any financial payments or other benefits from any commercial entity related to the subject of this article.
